# The Crest Phenotype in Chicken Is Associated with Ectopic Expression of *HOXC8* in Cranial Skin

**DOI:** 10.1371/journal.pone.0034012

**Published:** 2012-04-13

**Authors:** Yanqiang Wang, Yu Gao, Freyja Imsland, Xiaorong Gu, Chungang Feng, Ranran Liu, Chi Song, Michèle Tixier-Boichard, David Gourichon, Qingyuan Li, Kuanwei Chen, Huifang Li, Leif Andersson, Xiaoxiang Hu, Ning Li

**Affiliations:** 1 State Key Laboratory for Agrobiotechnology, China Agricultural University, Beijing, China; 2 Department of Medical Biochemistry and Microbiology, Uppsala University, Uppsala, Sweden; 3 Jiangsu lnstitute of Poultry Science, Yangzhou, China; 4 INRA, AgroParisTech, UMR1313 Animal Genetics and Integrative Biology, Jouy-en-Josas, France; 5 INRA, UE1295 PEAT, Nouzilly, France; 6 Department of Animal Breeding and Genetics, Swedish University of Agricultural Sciences, Uppsala, Sweden; Central China Normal University, China

## Abstract

The Crest phenotype is characterised by a tuft of elongated feathers atop the head. A similar phenotype is also seen in several wild bird species. Crest shows an autosomal incompletely dominant mode of inheritance and is associated with cerebral hernia. Here we show, using linkage analysis and genome-wide association, that *Crest* is located on the *E22C19W28* linkage group and that it shows complete association to the HOXC-cluster on this chromosome. Expression analysis of tissues from Crested and non-crested chickens, representing 26 different breeds, revealed that *HOXC8*, but not *HOXC12* or *HOXC13*, showed ectopic expression in cranial skin during embryonic development. We propose that Crest is caused by a cis-acting regulatory mutation underlying the ectopic expression of *HOXC8*. However, the identification of the causative mutation(s) has to await until a method becomes available for assembling this chromosomal region. Crest is unfortunately located in a genomic region that has so far defied all attempts to establish a contiguous sequence.

## Introduction

A feather-crested head is a prominent feature exhibited by several wild bird species, as well as varieties of several domesticated birds [1]. In chickens *Crest (Cr)* is an autosomal incompletely dominant mutation that causes a tuft of elongated feathers to sprout from the head, with homozygous individuals often exhibiting a more developed crest than heterozygotes. The phenotype shows a degree of sexual dimorphism, with males exhibiting more voluminous crests than females. Homozygosity for Crest has been associated with cerebral hernia that causes a malformation of the cranium [2], [3], [4], [5], [6].

The earliest known description of chickens with a Crest comes from the Roman author Claudius Aelianus, around the turn of the 3rd century AD. Archeological evidence indicates that crested chickens may have been among the earlier differentiated domestic varieties, having been discovered in a Roman era site in Britain that is presumed to have been active in the 4th century AD [7]. Scientific studies of the Crest phenotype go back more than 100 years. In 1906, a number of crosses involving the crested varieties Polish, Houdan and Silkie were set up by Davenport [8]. In 1928, Serebrovsky and Petrov published the first genetic linkage map of any domestic animal, including eight linkage groups in chickens, and the *Crest* locus was included in this map [9]. Further breeding experiments by various scientists have established *Crest* as belonging to linkage group II, which today is known to also contain the classical phenotypic loci *Fray* (*Fr*), *Dominant white* (*I*) and *Frizzle* (*F*) [10], [11], [12], [13] In 1934, Fisher reported that Crest was inherited in Mendelian proportions and was dominant over crestless head [5]. In 2004, the *Dominant white* locus was mapped to *E22C19W28* linkage group (*LGE22C19W28*) [14]. This placed linkage group II, including *Crest* in the molecular *LGE22C19W28* linkage group.

The chicken genome is composed of 38 pairs of autosomes and a pair of sex chromosomes. The autosomes are of vastly different sizes and are classified as either macro-chromosomes or micro-chromosomes. *LGE22C19W28* has not yet been assigned to a chromosome, but it is expected to reside on a micro-chromosome. For some micro-chromosomes and linkage groups, the chicken genome is still relatively deficient in markers. In the chicken consensus linkage map the length of *LGE22C19W28* was estimated to be 50 cM with five microsatellite markers and the *Dominant white* locus [15]. In March 2004 the first draft of the chicken genome was released, in which the physical map size of *LGE22C19W28* was about 70 Kb [16], [17]. In the Gallus_gallus-2.1 assembly, *LGE22C19W28* and *LGE50C23* were merged as *LGE22C19W28_E50C23*. As the map length of *LGE50C23* was 40 cM [15] the combined map length of *LGE22C19W28_E50C23* was then estimated at 90 cM, containing 900 kb ordered sequences and 190 kb random sequences.

In this study, we map *Crest* to the HOXC cluster in the *LGE22C19W28_E50C23* linkage group and report that the Crest trait is associated with ectopic expression of *HOXC8* in the cranial skin where the Crest develops.

## Results

### The Crest trait and the characteristics of the cranial feathers

**Figure 1:**
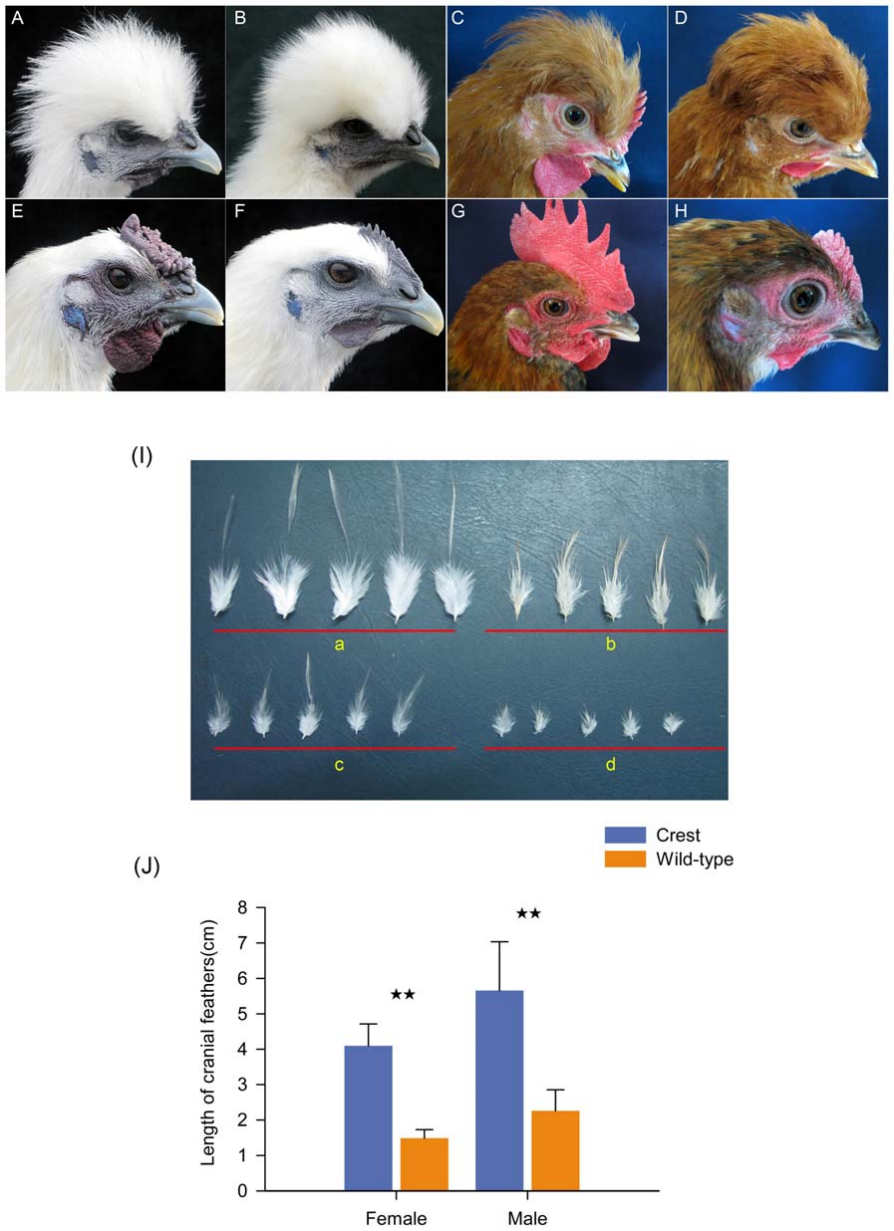
Crested and wild-type chickens. (A–D) Crest phenotype; (E–H) wild-type phenotype; (A and E) Silkie male; (B and F) Silkie female; (C) Chinese fatty chicken male; (D) Chinese fatty chicken female; (G) Chahua chicken male; (H) Chahua chicken female. (I) Overview of cranial feathers with gender indicated, sampled from 44 weeks old Silkie chickens. (a) Crested male; (b) Crested female; (c) Wild-type male; (d) Wild-type female. (J) There was a statistically significant difference in feather length between phenotypes. The number of feathers in each group obtained from three individuals were as follows: Crested male: 47; Crested female: 44; wild-type male: 100; wild-type female: 100 (**, P<0.01).

The Crest phenotype is characterised by a tuft of elongated feathers atop a chicken's head (Fig. 1A–H). This trait occurs in diverse chicken breeds from all over the world including the Appenzeller, Crèvecœur, Icelandic, Jinhuwu, Polish, Silkie, Sulmtaler, Sultan and Chinese fatty chicken. The appearance of the Crest depends on the length and shape of the cranial feathers. Individuals from a single Silkie population from southern China, segregating for Crest, were sampled for cranial feathers at the age of 45 weeks. Measurements revealed a significant difference in feather length between Crested and wild-type chickens (*P*<0.001) (Figs. 1I & 1J). The feather shape is also different between roosters and hens. The Crest of a rooster is composed of feathers that come to a sharp point whereas the Crest of a hen is composed of rounded feathers (Figs. 1C & 1D).

### Segregation of the Crest phenotype in the CAU F_2_ resource population

In this study, we constructed an F_2_ resource population derived from reciprocal crosses between the Crested Silkie and the non-crested White Plymouth Rock. All F_1_ individuals had the Crest phenotype consistent with a dominant inheritance. Over 3,000 F_2_ offspring were produced by inter-mating the F_1_ individuals. The observed ratio between Crested and non-crested F_2_ progeny (2324:789) did not deviate significantly from the expected 3:1 ratio (x^2^ = 0.10, d.f. = 1; *P* = 0.75).The result was fully consistent with previous studies showing that Crest is controlled by a single autosomal locus.

### Comparative alignment and extending the *LGE22C19W28_E50C23* linkage group

Comparative genomic data for chicken and human, based on DNA-DNA alignments, orthologous protein information and large-scale synteny data are provided in the Ensembl database [18]. The data indicate extensive conserved synteny between the human and chicken genomes. *LGE22C19W28_E50C23* of chicken corresponds to an orthologous region on human chromosome 12 (HSA12) near the 50 Mb region. The region between 45 Mb to 60 Mb on HSA12 shows patches of conserved synteny with other parts of the chicken genome including chicken chromosomes GGA1, GGA2, GGA7 and ChrUn_random, the latter consists of contigs that could not be localised to any specific chromosome. From position 46.34 Mb to 61.33 Mb on HSA12, there are 80 potential protein-coding genes orthologous between human and chicken, 38 of these have not been mapped to specific chromosomes, but are located on 29 contigs. Together these contigs comprise 739 kb of sequence and include the *HOXC* gene cluster which has an essential role in vertebrate development [19](Table S1).

We tried to extend the *LGE22C19W28_E50C23* linkage group by linkage analysis in the CAU F_2_ population using eight microsatellites. Five of these markers were derived from unassigned contigs for which the comparative data suggested that they may be located in *LGE22C19W28_E50C23* (Table S2). The marker *SKL0185*, belonging to contig15741, was located on *LGE22C19W28* in the Gallus_gallus-1.0 assembly (2004), but was missing in the Gallus_gallus-2.1 assembly (2006). The marker *SKL0019* is located in the same contig as marker *MCW0188*, which has been assigned to *LGE22C19W28* [15]. Three additional markers, *SKL0067*, *SKL0071* and *ROS0306* are from unassigned contigs showing sequence homology to HSA12. Three other markers. *SCN8A* [20], M*CW0317* and *SKL0052* are located on *LGE22C19W28_E50C23*, and were selected to anchor and build the linkage map. The linkage analysis conclusively demonstrated that all eight markers map to *LGE22C19W28_E50C23* (data not shown).

The markers were further analysed with respect to the *Crest* locus using a pedigree material comprising 88 individuals. *Crest* showed genetic linkage to all eight microsatellite markers, *SKL0019*, *MCW0317*, *SKL0185*, *SCN8A*, *SKL0071*, *SKL0067*, *ROS0306* and *SKL0052* (Table 1). The linkage analysis revealed no recombination between *SKL0071* and *Crest* (LOD score = 4.82), or between *Crest* and *SKL0067* (LOD score = 5.42).

**Table 1:** Two-point linkage analysis of the *Crest* locus in the CAU F_2_ population using eight microsatellite loci on chicken *LGE22C19W28_E50C23*.

Locus	Marker	Recombination Fraction	LOD Score
*Crest*	*SKL0019*	0.10	4.50
*Crest*	*MCW0317*	0.09	5.83
*Crest*	*SKL0185*	0.11	5.76
*Crest*	*SCN8A*	0.06	7.48
*Crest*	*SKL0067*	0	5.42
*Crest*	*SKL0071*	0	4.82
*Crest*	*ROS0306*	0.05	8.04
*Crest*	*SKL0052*	0.06	6.08

### Genome wide association study (GWAS) and fine mapping of *Crest*

278 individuals of the CAU resource population, belonging to 15 full-sib pedigrees, were used for a whole genome association study and fine mapping analysis. A total of 43,131 SNPs were used together with the microsatellite marker *HOXC8-ssr* and the SNP *HOXC8-3end* that are located upstream and downstream of *HOXC8*, respectively. These two markers were identified by analysis of a bacterial artificial chromosome (BAC) from the HOXC cluster region and the polymorphisms were detected by analysing Crested and wild-type individuals (data not shown).

**Figure 2:**
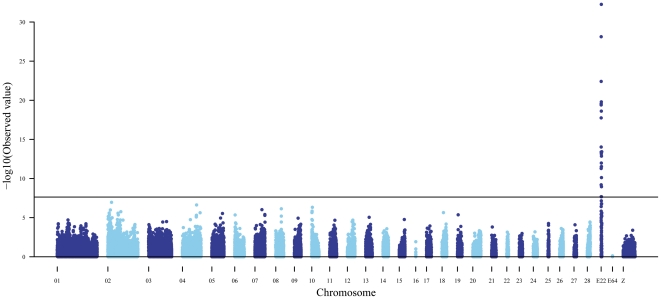
Manhattan plot of genome-wide association analysis for the Crest phenotype in chicken. The x-axis shows the physical position of the SNPs by chromosome, and the y-axis shows -log10(p-values). A cutoff value of 7.63 declares a genome-wide highly significant association (P<0.001) with a Bonferroni correction. The microsatellite marker *HOXC8*–*ssr that* had the most significant *p*-value is located on *LGE22C19W28* by *HOXC8*.

All the genotyping information was analysed in genome-wide association study (GWAS) and linkage analysis. We found that *HOXC8-ssr*, *HOXC8-3end* and *HOXC11* all showed highly significant significant linkage to *Crest* (*P* = 5.5×10^−33^) with no recombination event detected (Fig. 2, Table S3 and S4). This suggests that the mutation underlying the Crest phenotype might be located within the HOXC cluster, in the vicinity of *HOXC8*. The HOXC cluster is not properly assembled in the chicken genome but the human HOXC cluster contains eight HOXC genes (*HOXC4-6* and *HOXC8-13*) in tandem over a 100 kb region on HSA12.

In total 117 genetic markers were used to construct a linkage map of *LGE22C19W28_E50C23*, 18 of these markers were derived from unassembled regions that we now mapped to this linkage group.

### Fine mapping of *Crest* using the INRA resource population

An INRA resource population segregating for a number of traits derived from European chicken breeds, including *Crest* was utilised as a population of Crested birds distantly related to Chinese Crested breeds. An F_1_ cross using two Cr/cr^+^ males from this population, crossed with 16 inbred cr^+^/cr^+^ White Leghorn females yielded 383 hatched chicks, 186 Crested, 196 non-crested and one whose phenotype could not be determined.

A panel of four microsatellites and 11 SNPs were genotyped for mapping *Crest* in this population. The markers were selected for their association with Linkage group II, *LGE22C19W28_E50C23* or *HOXC8* (Table S5 and S6).

SNPs in the immediate vicinity of *HOXC8*, one in an intron and another in the 3′ downstream region showed no recombination with *Crest* in this material (Table 2).

**Table 2:** Two-point linkage analysis of the *Crest* locus in the INRA resource population and 15 marker loci.

Locus	Marker	Recombination fraction	LOD score	Method	Location^a^	Contig
*Crest*	*MCW317*	0.09	61.37	Microsatellite	E22C19W28_E50C23	Contig633.3
*Crest*	*SCN8A*	0.08	47.58	Microsatellite	E22C19W28_E50C23	Contig164.44
*Crest*	*E22-164.33*	0.07	69.87	TaqMan	E22C19W28_E50C23	Contig164.33
*Crest*	*E22-164.30*	0.07	70.14	TaqMan	E22C19W28_E50C23	Contig164.30
*Crest*	*HOXC-SCF2*	0.04	18.78	Pyrosequencing	Un_random/BAC WAG-73F24	Contig1453.1
*Crest*	*HOXC8-INTR*	0	112.59	TaqMan	WAG-73F24	
*Crest*	*HOXC8-3end*	0	110.18	TaqMan	Un_random/BAC WAG-73F24	Contig11389.1
*Crest*	*E22-9570*	0.02	96.58	TaqMan	Un_random	Contig9570.1
*Crest*	*HOXC-SCF1*	0.08	6.54	Pyrosequencing	Un_random/BAC WAG-73F24	Contig18292.1
*Crest*	*MYG1*	0.04	83	TaqMan	Un_random/Linkage group II	Contig303.3
*Crest*	*ROS0306*	0.04	69.63	Microsatellite	Un_random/Linkage group II	Contig303.14
*Crest*	*MCW188*	0.06	31.17	Microsatellite	Linkage group II	
*Crest*	*E22-663.2*	0.23	24.36	TaqMan	E22C19W28_E50C23	Contig663.2
*Crest*	*E22-186.17*	0.24	22.54	TaqMan	E22C19W28_E50C23	Contig186.17
*Crest*	*E22-186.52*	0.25	20.58	TaqMan	E22C19W28_E50C23	Contig186.52

The lower LOD scores reported for *HOXC-SCF1* and *HOXC-SCF2* reflect the fact that only individuals with a recombination event between MCW317 and E22-186.2 were typed for these markers.

a Un_random means markers from unassigned contigs; WAG_73F24 represents markers derived from sequences found in BAC WAG_73F24.

### Expression analysis of typical *HOXC* genes

In mice, *Hoxc12* is reported to have an expression pattern limited to the epidermal cells of the hair follicle [21]. Overexpression of *Hoxc13* in the hair follicles of transgenic mice resulted in baldness accompanied by pathologically dry, flaky and scaly skin. Interestingly, *Hoxc13* knockout mice also showed a phenotype resulting in baldness [22], [23]. Chicken *HOXC8* is reported to be expressed during embryonic development in the dorsal dermal and epidermal cells during the first stage of feather morphogenesis [24]. Because mammalian hair and avian feathers are similar structures, both being derivatives of ectoderm, these three *Hox* genes (*HOXC8*, *HOXC12* and *HOXC13*) were chosen as candidate genes for the *Crest* locus.

**Figure 3:**
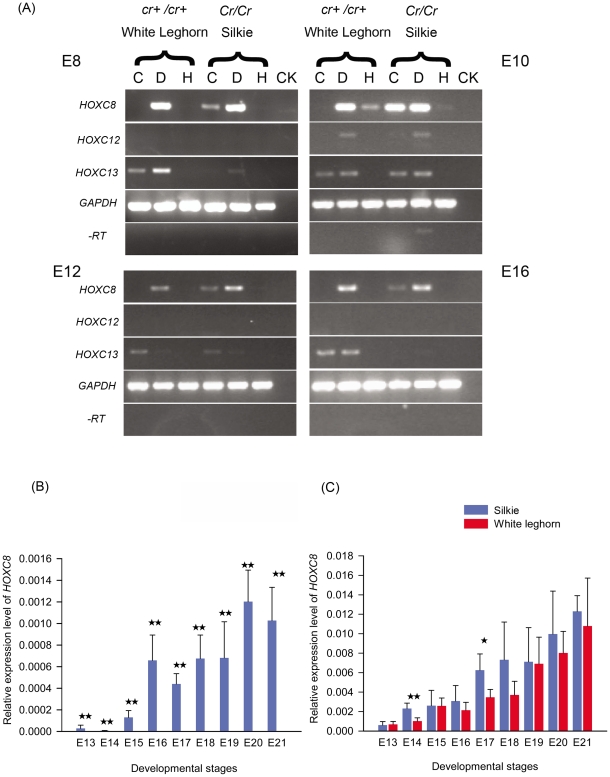
Expression analysis of HOXC genes in Silkie (crested) and White Leghorn (non-crested) chickens. (A) *HOXC8*, *HOXC12* and *HOXC13* genes were detected by RT-PCR in three tissues (cranial skin, dorsal skin and heart) of Silky and White Leghorn chickens at four developmental stages (E8, E10, E12, E16). C: Cranial skin; D: Dorsal skin; H: Heart. *GAPDH* was used as a positive and normalised control. -RT indicates a reaction without reverse transcriptase. CK indicates a reaction with water as the template. (B)&(C) q-PCR results for *HOXC8* expression level in cranial skin (B); and in dorsal skin (C) during chicken developmental stages E13 to E21 comparing Crested and non-crested chickens. No *HOXC8* expression was detected in cranial skin from non-crested birds. (*, *P*<0.05; **, *P*<0.01).

**Figure 4:**
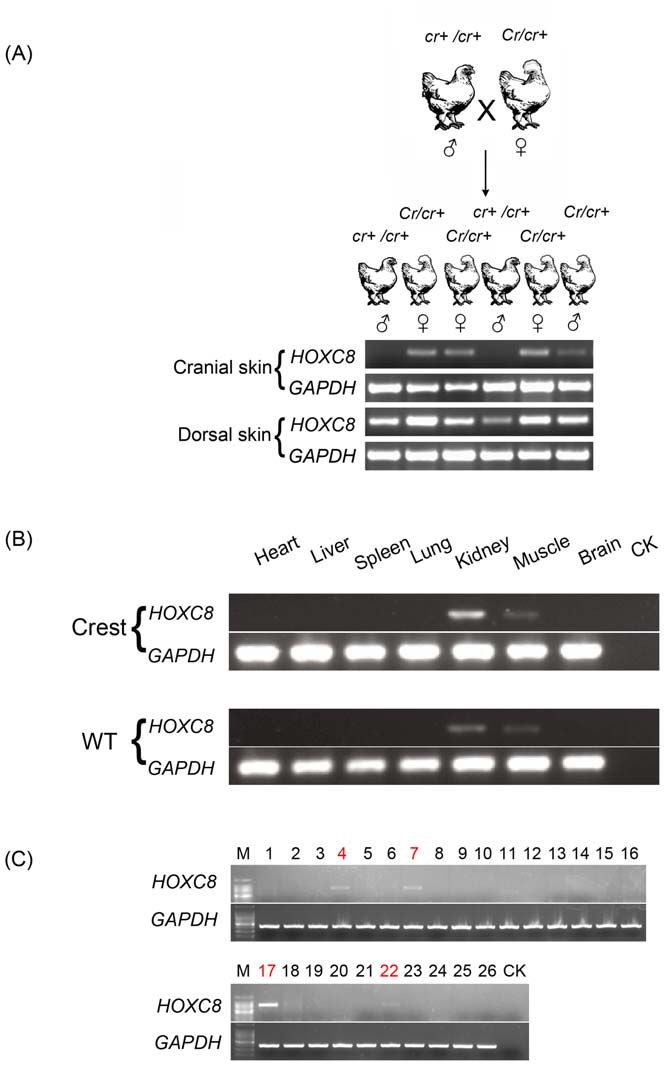
RT-PCR analysis of *HOXC8* in different tissues in various chicken breeds. (A) RT-PCR analysis in skin samples from the progeny of a heterozygous *Cr*/*cr^+^* chicken. (B) RT-PCR of seven tissues from *Crested* and wild-type Silkie chickens. Samples were collected from two individuals at 12 weeks of age. -RT indicates a reaction without reverse transcriptase. CK indicates negative control. (C) RT-PCR results for the cranial skin in adult individuals of 26 Chinese local chicken breeds. Breeds are as follows: 1. Lu Yuan; 2. Gu Shi; 3. Hu Xu; 4.Chinese Fatty Chicken; 5. Xian Ju; 6. Da Gu; 7. Silkie; 8. Dou Chicken; 9. Green Egg Chicken; 10. Wen Chang; 11. You Xi Ma; 12. Ai Jiao Huang; 13. Shou Guang; 14. An Ka; 15. Bian Chicken; 16. White Ear; 17. Kuai Da Silkie; 18. Cha Hua; 19. Yin Xing Bai; 20. Chong Ren Ma; 21. Wa Hui; 22. Jin Hu Wu; 23. Tibet Chicken; 24. Shi Qi Za; 25. Black Lang Shan; 26. Qing Yuan Ma; CK: Negative control. Numbers in red indicate chicken breeds with *Crest* phenotype and black numbers wild-type breeds (Table S12).

Four embryonic developmental stages were chosen (E8, E10, E12 and E16) for expression analysis using Crested (Silkie) and wild-type (White Leghorn) embryos. RT-PCR analysis revealed that only *HOXC8* showed phenotype-specific expression in the cranial skin of Crested chickens (Fig. 3A). The same expression difference was confirmed in 26 local Chinese chicken breeds, 4 of which are fixed for Crest and 22 of which are fixed for wild-type, as well as in a full-sib Silkie pedigree segregating for *Crest* (Figs. 4A&4C, Table S7). Expression profiling was also performed in other tissues from a Crested and a wild-type Silkie, both at 12 weeks of age. Expression analysis by quantitative PCR (q-PCR) analysis using different skin tissues from embryonic stage E13 to E21 showed a trend of increased expression as the embryo developed (Fig. 3B&C). Apart from the skin tissues, *HOXC8* expression was only detected in kidney and muscle, indicating the gene is not a widely expressed transcription factor in chicken (Fig. 4B). Northern blot and whole mount *in situ* hybridisation was also attempted but we failed to obtain a clear positive signal (data not shown). Based on the q-PCR results, we suggest that low expression levels of *HOXC8* could explain the poor signal.

**Figure 5:**
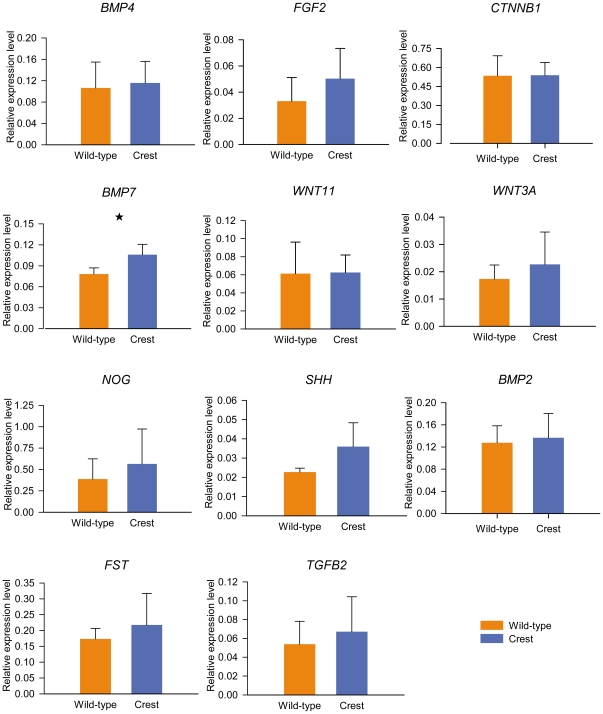
RT-PCR analysis of genes involved in feather development in cranial skin from Crested and wild-type chickens (*, *P*<0.05). *GAPDH* was used as a normalising control. Relative expression levels were obtained by the 2^−ΔΔCT^ method.

To detect downstream targets that might function in a network with HOXC8 during feather development, we examined the expression levels of 11 genes, *BMP4*, *BMP2*, *FGF2*, *CTNNB1*, *WNT11*, *NOG*, *WNT3A*, *SHH*, *FST*, *BMP7* and *TGFB2* by q-PCR in cranial skin tissue in 12 weeks old adult Crested and wild-type Silkies. We found that of these 11 genes, only *BMP7* showed a significant difference in expression between the different genotypes (*P*<0.05) (Fig. 5). It could be speculated that *HOXC8* and *BMP7* might act together in the same pathway that affects feather growth. Loss-of -function and gain-of -function analysis would be necessary for testing this hypothesis.

### Characterisation of chicken *HOXC8* by sequencing

**Figure 6:**
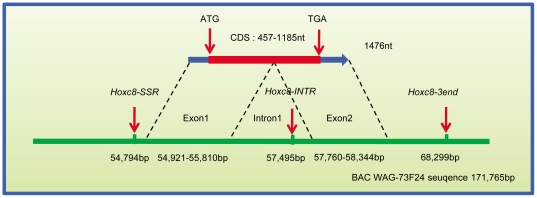
Gene structure of chicken *HOXC8*. The two most significant genetic markers in the GWAS of *Crest* are indicated.

The genomic sequence of *HOXC8* (Gene ID: 395711) in chicken was only 405 bp long (NW_001477359) in the Gallus_gallus-2.1 chicken genome release, whereas the chicken *HOXC8* mRNA sequence is 1476 nt (NM_204893). A White Leghorn BAC clone, WAG-73F24, containing *HOXC8* and *HOXC13* was identified in a previous study [25]. Therefore we sequenced this BAC clone, obtaining 171,765 bp of sequence, from which 86 scaffolds were assembled. *HOXC8* and *HOXC6* were located in scaffold 3, *HOXC11*, *HOXC10*, *HOXC9* in scaffold 2 and *HOXC13* in scaffold 1. We found that the chicken *HOXC8* gene is made up of two exons and one intron (Fig. 6). The result is consistent with the two-exon structure of human *HOXC8* (Gene ID: 3224) and mouse *Hoxc8* (Gene ID: 15426).

Due to the high rate of repetitive and polynucleotide sequence structures in the *HOXC8* genomic region, there are still 20 unfilled gaps in scaffold 3 after our BAC sequencing effort. This has made it impossible to completely resequence *HOXC8* and its flanking region with a PCR-based approach in the search for candidate causative mutations.

## Discussion

### Crest is associated with ectopic expression of *HOXC8*

This study has provided conclusive evidence that the *Crest* mutation is located in the linkage group *LGE22C19W28_E50C2*3 in the near vicinity of *HOXC8*. In the CAU F_2_ population no recombination was detected between Crest and two genetic markers, *HOXC8-ssr* and *HOXC8-3end*, that are in the immediate vicinity of *HOXC8* for 338 and 221 informative meioses respectively. The linkage mapping for the INRA population shows the same association. This result contradicts a previous report based on fluorescent in situ hybridisation (FISH) that indicated that the HOXC cluster is located on chicken chromosome 1 and also the NCBI GenBank description about the position of chicken *HOXC8* (Gene ID: 395711, at NW_003778389.1, from 237 bp to 1871 bp) [26]. We have also demonstrated that the Crest phenotype is associated with ectopic expression of *HOXC8* in cranial skin during development which appears to be a reasonable explanation for the atypical feather growth on the heads of Crested chickens. We therefore propose that Crest is caused by a cis-acting regulatory mutation that underlies the ectopic expression of *HOXC8* that we have confirmed in four different breeds of Crested chickens. Such a regulatory mutation may be located in a region corresponding to one of the *HOXC8* regulatory elements that have been defined in other species, including a microRNA binding site, an early enhancer element, and a long-range regulatory sequence in the downstream region [27], [28], [29]. However, we have not been able to identify the causative mutation as it has not yet been possible to assemble this chromosomal region. The *LGE22C19W28_E50C23* linkage group is most likely located on a microchromosome and several of these have been extremely difficult to assemble due to high GC content and high density of various forms of repetitive sequences. The HOXC cluster is not included in the current genome assembly for chicken. We were not able to assemble it using BAC sequencing and we have failed to use long-range PCR to connect contigs established by BAC sequencing. Therefore, the molecular characterisation of the *Crest* causative mutation has to await until a method for assembling the dark side of the chicken genome (primarily microchromosomes) has been established.

### Chicken *HOXC8* and feather development

Hox homeodomain transcription factors play a crucial role in animal embryonic development. They regulate numerous developmental pathways and are involved in cellular processes such as organogenesis, cellular differentiation, cell adhesion and migration, cell cycle and apoptosis [30]. Morphological diversity between different groups of animals is under considerable influence from Hox genes [31], [32], [33].

Feather development involves a series of interactions between epithelium and mesenchyme, resulting in the formation of a specialised keratinous appendage, the feather. The formation of a structure as complex as a feather necessitates a complex pattern of interactions and signals. Among the signaling molecules that have essential roles in feather development are bone morphogenetic proteins 2 and 7 (*BMP2 and BMP7*), transforming growth factor β2 (*TGFB2*), *β-catenin* (*CTNNB1*), fibroblast growth factors 2 and 4 (*FGF2* and *FGF4*), fibroblast growth factor receptors (*FGFRs*), noggin(*NOG*), Follistatin(FST), *WNT3A*, *WNT7A*, *WNNT11*, sonic hedgehog (*SHH*), Delta-1 and epidermal growth factor (*EGF*) [34], [35].

*HOX* genes are considered to be master transcription factors, with a function at the top of a genetic hierarchy, exerting great control over a particular pathway. Direct targets of *Hoxc8* in mice include neural cell adhesion molecule (*Ncam*), cadherin 11 (*Cdh11*), osteopontin (*Opn*), pigment epithelium-derived factor (*Pedf*) and zinc finger protein regulator of apoptosis 1 (*Zac1*). These five *HOXC8* regulated genes are involved in the *Wnt*, *BMP*, and *FGF* signalling pathways, all of which have been shown to be involved in feather morphogenesis [36].

In this study, we found that *HOXC8* was the only candidate gene examined with a strikingly altered expression pattern, which may directly influence the development of feathers, especially in terms of the morphology of the cranial feathers and thus cause the Crest phenotype in chicken.

### An exception to the traditional Hox colinearity model

*Hox* genes are present in distantly related groups of animals, including worms, insects, fish, frogs, birds and mammals, and are thought to have arisen in a bilateral ancestor to these groups. Comparison of the composition of *Hox* gene clusters in various animals indicates that this ancestor had a cluster of at least eight *Hox*-class genes The relative order of the genes in *Hox* clusters is in general conserved between species. This in turn tends to dictate a spatially and/or temporally colinear pattern of expression that progresses according to the chromosomal position of the genes in the cluster [37], [38].

Colinearity was originally described in *Drosophila* [38], and has since been widely observed in animals exhibiting an anterior-to-posterior axial polarity. Some exceptions to the colinearity of Hox gene expression have been discovered. Murine *Hoxc13* is according to the model of colinearity a posterior gene, associated with the hindquarters, but it was found to be expressed in hair follicles all over the body, including those of the whiskers, as well as in the tongue. This was the first report of an exception to the traditional model of colinearity [23], [39].

Spatial colinearity has been reported to occur for several Hox genes expressed in developing chicken skin (*HOXB4*, *HOXA7* and *HOXC8*). Other Hox genes, for instance the HOXD cluster, did not show such colinearity in developing chicken skin [40]. In our study, the *HOXC8* gene showed a tissue-specific expression pattern in cranial skin in birds carrying the *Crest* mutation. Considering that *HOXC8* is natively expressed in dorsal skin during the initial stages of feather follicle formation [24] and that the genomic location of *HOXC8* is medial in the HOXC cluster it could be argued that the expression of chicken *HOXC8* in cranial skin of *Crest* mutants is also evidence for an exception to the colinearity model. Yet we cannot at present exclude the possibility that a chromosomal rearrangement has occurred on the Crest chromosome. If such a rearrangement would eliminate HOXC8 expression from its native expression area, a more severe phenotype might arise, due to the importance of maintaining normal Hox gene expression for developmental patterning in bilateral animals.

That *HOXC8* is natively expressed in the dorsal dermis around embryonic day 8 (E8) [24] is something that may tie in with the sexual dimorphism in feather shape of the Crest. It is analogous to the sexual dimorphism of feather shape in the upper tail coverts, or saddle, of the domestic chicken. Saddle feathers in roosters are elongate and pointed, not dissimilar to the hackles and Crest feathers. The same feathers in hens are shorter and rounded (Figs. 1C & 1D). This gives rise to the suggestion that the ectopic expression of *HOXC8* associated with *Crest* may reprogram the cranial dermis to act as if it were dorsal dermis.

## Materials and Methods

### Ethics statement

All the chickens at INRA experimental farm were fed and sacrificed according to national regulations and standards of animal welfare. The farm is registered by the ministry of Agriculture with the license number B37-175-1 for animal experimentation.

All the chickens at China Agricultural University (CAU) were fed and sacrificed according to local standards of animal welfare issues. The study was approved by the animal welfare committee of China Agricultural University with approval number XK257.

### CAU Chicken resource population and DNA sample preparation

The CAU chicken resource population was derived using an F_2_ design from reciprocal crosses between Silkie and White Plymouth Rock chickens. The Silkie line was chosen as one of the native lines that had been maintained for generations as a closed population at CAU. Silkie is homozygous mutant *Crest* (*Cr*/*Cr*) while White Plymouth Rock chickens are homozygous wild-type (*cr^+^*/*cr^+^*). There were 26 males and 160 females in the F_0_ population (comprising 58 Silkies and 128 White Plymouth Rock chickens). A reciprocal cross produced the F_1_ generation. The F_2_ generation was produced by mating 33 F_1_ males and 165 F_1_ females, and over 3,000 F_2_ offspring were hatched. Blood samples were collected from all F_0_, F_1_ and F_2_ individuals at 12 weeks of age by superficial venipuncture of a wing vein. DNA was prepared by standard procedures [41].

### Genotyping and primary linkage analysis by microsatellite markers

Microsatellites were PCR amplified in a total volume of 15 µl containing 40 ng genomic DNA, 1.5 mM MgCl_2_, 50 mM KCl, 10 mM Tris·HCl (pH = 8.3), 0.1% Triton X-100, 200 mM dNTPs, 0.01% gelatine, 1 U TaqDNA polymerase (Promega Corporation, Madison, WI, USA) and 8 pmol of each primer, one of the primers in each pair was fluorescently labelled. Following an initial incubation at 94°C for 5 min, amplification reactions were performed for 35 cycles each with denaturing at 94°C for 30 s, annealing at 60°C for 30 s, and extension at 72°C for 1 min, and a final elongation step at 72°C for 10 min. The PCR products were electrophoresed in 6% polyacrylamide gel using an ABI377 sequencer (Applied Biosystems, Foster City, CA, USA). Fragment sizes were determined by using the GeneScan 3.1 fragment analysis software (Applied Biosystems) and allele identification was performed using the Genotyper 2.1 software (Applied Biosystems).

Genotype data from four full-sib families, comprising 88 birds, were analysed for eight microsatellites and the *Crest* locus (primers are listed in Table S2). Linkage analysis was performed using the Crimap software [42]. Initially, the Two-point option of CRI-MAP was used and the markers were grouped by two-point value. Then the order of the different loci was checked using the FLIPS option.

### Sequencing of a BAC clone containing *HOXC* genes

*De novo* sequencing data for the White Leghorn BAC clone WAG-73F24 were obtained by sub-cloning and sequencing with Illumina genome analyser (Illumina, San Diego, USA) in one lane [25]. All primary sequence data were assembled by the BGI bioinformatics service (Shenzhen, China). All assembled data for the BAC clone have been submitted to GenBank with accession number JN129278.

### Genotyping the markers used in GWAS and fine mapping analysis

A genotyping experiment using Illumina 60 K chicken SNP Beadchip was outsourced to the Illumina-certified service provider, DNA LandMarks Inc., Canada. Quality control was performed in GenomeStudio. One sample was excluded due to a low call rate (<95%). 14,997 SNPs were removed with the following metrics: low call frequency (<95%), low heterozygosity intensity and cluster separation value (<0.4), heritability or replication error, and low minor allele frequency (<0.1). A total of 42,639 SNPs were used in the subsequent analysis.

In addition, seven SNPs (*rs14741264*, *rs14689665*, *rs16019364*, *rs16687544*, *rs16687551*, *rs16710870* and *rs16714644*) were analysed using SNPlex assays (Applied Biosystems, Foster city, USA). *rs14741264* is located in *HOXC11* (GeneBank ID:430698) and it was also aligned to our *de novo* BAC sequence.

The *HOXC8-3end* SNP was genotyped using pyrosequencing (Biotage, Sweden). Primers were designed using the Assay Design software. The microsatellite marker *HOXC8-ssr* was amplified using HotStarTaqPlus DNA Polymerase kit (Qiagen, Cat. no. 203603, Germany). The PCR products were denatured for 5 min at 94°C before electrophoresis in POP-7™ polymer (Applied Biosystems) on an ABI3130xl sequencer (Applied Biosystems). The results were analysed with ABI Genemapper 4.1 software. There were two alleles (314 bp, 322 bp) according to the length of PCR products.

### Genome-wide association study and linkage analysis for *LGE22C19W28*

Statistical tests for GWAS were carried out using the epiSNP2 computer package (version 3.4), which implemented the extended Kempthorne model that allows linkage disequilibrium between SNPs and Hardy-Weinberg disequilibrium of each SNP [43], and used a two-step generalised least squares analysis that detected association accounting for sample relationship [44]. To eliminate some spurious associations, a *P*-value threshold of 2.3×10^−8^ was considered to be genome-wide significance (*P*<0.001) with a Bonferroni correction.

Fine mapping for linkage analysis was conducted with the same markers as used in the GWAS. The linkage map for the *LGE22C19W28* linkage group was constructed on a 64-bit Linux system with Crimap software (version 2.503) which was kindly provided by Dr. Jill Maddox. Markers with inheritance errors or double recombinant error caused by genotyping were checked and excluded manually. All unassigned SNPs were mapped to *LGE22C19W28* using Crimap “two-point” option, these SNPs were added to the new linkage group if they showed significant linkage with markers from *LGE22C19W28* (LODscore>3). The orders of all markers in the new *LGE22C19W28* linkage map were corrected by “all” and “flips” options.

### Tissue collection, RT-PCR and quantitative real-time RT-PCR

Tissues samples (heart, liver, spleen, lung, kidney, heart, brain, muscle, cranial skin and dorsal skin) collected from chicken embryos or adult chickens were immediately stored in liquid nitrogen. Total RNA was extracted using Trizol (Tiangen, Beijing, China) or RNA RNeasy Mini Kit (Qiagen, Cat.no. 74104). The RNA preparations were treated with RNase free DNase I to remove potentially contaminating DNA. First-strand cDNA was synthesised using 2 µg RNA with an oligo-dT primer. RT-PCR Primers were designed using the Primer3 web tool (http://frodo.wi.mit.edu/primer3/).

Samples were run in triplicate on ABI Prism 7900 HT Sequence Detection System (Applied Biosystems) according to the manufacturer's protocol. Four individuals of each embryonic stage were used for each breed in q-PCR experiments to detect the expression level of *HOXC8*. *GAPDH* was used to normalise each sample. Primers (Table S7) were designed using the Primer Express Software (Applied Biosystems). q-PCR for *HOXC8* from E13 to E21 was conducted by Taqman Universal PCR Master Mix (Applied Biosystems) using fluorescent marked probe at the 5′ end of the probe. Separately, plasmids containing *HOXC8* and *GAPDH* gene fragments were diluted to a concentration of 10^−8^, 10^−7^, 10^−6^, 10^−5^ or 10^−4^ to make standard curve. Each 15 µl q-PCR reaction was made up of 7.5 µl master mix, 150 nM of each primer, 75 nM of each probe and 1 µl cDNA as template.

q-PCR of eleven genes (*BMP4*, *FGF2*, *BMP2*, *CTNNB1*, *BMP7*, *WNT11*, *NOG*, *WNT3A*, *SHH*, *FST* and*TGFB2*) for pathway analysis was conducted in triplicate by the 2^−ΔΔCT^ method [45]. *GAPDH* was used as a normalisation control. Eight individuals (four for each phenotype, Crest and wild-type) were used. Each 15 µl q-PCR reaction was made up of 7.5 µl master mix, 150 nM of each primer and 1 ul cDNA as template.

Statistical analysis was performed using SPSS software (version 17.0) for a two-sample *t*-test on the average of each of the triplicates.

### INRA population and linkage analysis thereof

The resource population at INRA is bred from numerous European chicken breeds and segregates for various traits found in European fowl populations. Two roosters from this population, both heterozygous for *Crest* were mated to inbred non-crested White Leghorn hens, eight females for each rooster, 16 hens in total. Approximately 200 chicks sired by each rooster hatched. Chicks were produced in two batches hatched three weeks apart. Phenotypes for Crest were scored by visual examination of each bird at the age of 46 or 47 days, depending on the batch. Blood sample for DNA extraction was collected from the wing vein of each chicken on the day of phenotypic recording.

Linkage analysis for Crest in this population was performed by genotyping a panel of microsatellite markers and SNPs via Pyrosequencing and TaqMan genotyping assays. 5′-fluorescent M13 primers combined with a primer with an M13 tail were used in amplification of microsatellite markers. The PCR products were denatured for 2 min before electrophoresis in 4% polyacrylamide gels using a MegaBACE capillary instrument (Amersham Bio- sciences, Uppsala, Sweden). The results were analysed with Genetic Profiler software (Amersham Biosciences). Pyrosequencing was performed using the SNP Reagent Kit protocol (Pyrosequencing AB, Uppsala, Sweden). 5′-biotinylated M13 primers combined with a primer with an M13 tail were used to allow capture of single stranded products onto avidin-coated beads. Custom Taqman@ SNP Genotyping Assays (Applied Biosystems) were used for nine SNPs, run according to manufacturer's instructions on a 7900HT Fast Real-Time PCR system (Applied Biosystem). Genotyping calls were made with SDS 2.3 software (Applied Biosystems). Primers were designed with the Primer3Plus webtool (http://www.bioinformatics.nl/cgi-bin/primer3plus/primer3plus.cgi); see Table S5, S6, S8, S9, S10 and S11 for further method information. Linkage analysis for the INRA population was performed with Crimap as previously described for the CAU population.

## Supporting Information

Table S1 **A list of the potential orthologous genes between chicken and human on HSA 12 from 46 Mb to 60 Mb.**
(DOC)

Table S2 **Primers used for genotyping in CAU population.**
(XLS)

Table S3 **Two-point linkage analysis for fine mapping of Crest in CAU F2 population and genetic markers on chicken LGE22C19W28.**
(XLS)

Table S4 **Marker alleles associated with the Crest haplotype derived from Silkie chickens.** All markers showed 1‰ genome-wise significance.
(XLS)

Table S5 **Primers used for analysis of INRA population microsatellites and pyrosequenced SNPs.**
(XLS)

Table S6 **Primers used for amplification and analysis of INRA population SNPs assayed with a TaqMan Genotyping Assay.**
(XLS)

Table S7 **Primers used for RT-PCR and q-PCR.**
(XLS)

Table S8 **Amplification conditions used for INRA population microsatellites.** M13 primers (5′-CACGACGTTGTAAAACGAC-3′) were variously labeled with the fluoresent dyes Hex, Fam and Tet.
(XLS)

Table S9 **PCR conditions for INRA population microsatellite analysis.**
(XLS)

Table S10 **Amplification conditions used for INRA population microsatellites.** M13 primer (5′-CACGACGTTGTAAAACGAC-3′) was labeled with biotin.
(XLS)

Table S11 **PCR conditions for INRA population pyrosequencing reaction.**
(XLS)

Table S12 **Phenotypic information for 26 different chicken breeds.**
(XLS)
