# Supplementary material for: The Crest Phenotype in Chicken Is Associated with Ectopic Expression of HOXC8 in Cranial Skin
Source: PLoS One. 2012 Apr 13;7(4):e34012. doi: 10.1371/journal.pone.0034012 (PMC3326004; doi:10.1371/journal.pone.0034012)
Supplement: Table S1 — A list of the potential orthologous genes between chicken and human on HSA 12 from 46 Mb to 60 Mb. (DOC) [file pone.0034012.s001.doc]

| Human Proteins | the position on HSA12 | Chicken Homologues | Chicken Chromosome | the position on GGA | Contig | the length of contig (bp) |
| --- | --- | --- | --- | --- | --- | --- |
| VDR | 46.52 Mb | NM_205098.1 | Un_random | 31.43 Mb | Contig303 | 91159 |
| TMEM106C | 46.64 Mb | LOC426186 | Un_random | 31.47 Mb | Contig303 | 91159 |
| SENP1 | 46.72 Mb | LOC426185 | Un_random | 31.49 Mb | Contig303 | 91159 |
| PFKM | 46.79 Mb | NM_204223.1 | Un_random | 31.51 Mb | Contig2326 | 8981 |
| ASB8 | 46.83 Mb | NP_001012954.1 | Un_random | 31.52 Mb | Contig2326 | 8981 |
| C1QL4 | 48.01 Mb | LOC430993 | Un_random | 24.35 Mb | Contig8677 | 4139 |
| NP_079178.2 | 48.03 Mb | LOC430880 | Un_random | 5.37 Mb | Contig1196 | 13038 |
| ACCN2 | 48.74 Mb | ACCN2 | Un_random | 61.79 Mb | Contig9308 | 3488 |
| TFCP2 | 49.77 Mb | TFCP2_CHICK | Un_random | 58.85 Mb | Contig846 | 14497 |
| ELA1 | 50.01 Mb | Q9PRV2_CHICK | Un_random | 934.33 Kb | Contig1032 | 9913 |
| GALNT6 | 50.03 Mb | NP_001026749.1 | Un_random | 57.64 Mb | Contig809 | 16553 |
| SLC4A8 | 50.07 Mb | LOC776010 | Un_random | 30.88 Mb | Contig2963 | 7645 |
| ANKRD33 | 50.57 Mb | LOC430112 | Un_random | 22.67 Mb | Contig2175 | 10104 |
| MFSD5 | 51.93 Mb | MFSD5 | Un_random | 57.77 Mb | Contig8122 | 4057 |
| ESPL1 | 51.95 Mb | ESPL1 | Un_random | 12.09 Mb | Contig1512 | 12875 |
| PFDN5 | 51.98 Mb | PFDN5 | Un_random | 12.10 Mb | Contig1512 | 12875 |
| C12orf10 | 51.98 Mb | LOC426187 | Un_random | 31.43 Mb | Contig303 | 91159 |
| AAAS | 51.99 Mb | AAAS | Un_random | 31.42 Mb | Contig303 | 91159 |
| PCBP2 | 52.13 Mb | LOC426023 | Un_random | 26.59 Mb | Contig2499 | 7067 |
| ATF7 | 52.20 Mb | XR_026691.1 | Un_random | 38.35 Mb | Contig397 | 49426 |
| HOXC13 | 52.62 Mb | HOXC13 | Un_random | 4.24 Mb | Contig1151 | 19423 |
| HOXC12 | 52.63 Mb | LOC429400 | Un_random | 36.33 Mb | Contig3677 | 5138 |
| HOXC11 | 52.65 Mb | LOC430698 | Un_random | 11.04 Mb | Contig1453 | 12790 |
| HOXC10 | 52.67 Mb | LOC770471 | Un_random | 29.26 Mb | Contig2791 | 5356 |
| HOXC9 | 52.68 Mb | HXD9_CHICK | Un_random | 20.05 Mb | Contig1993 | 8221 |
| SMUG1 | 52.86 Mb | LOC431025 | Un_random | 51.42 Mb | Contig6507 | 3869 |
| PDE1B | 53.23 Mb | XR_026702.1 | Un_random | 53.72 Mb | Contig7078 | 4097 |
| ENSG00000205332 | 53.87 Mb | ENSGALG00000010660 | Un_random | 18.03 Mb | Contig188 | 314702 |
| HCC1_HUMAN | 54.44 Mb | NP_001026495.1 | Un_random | 40.82 Mb | Contig436 | 39578 |
| ORMDL2 | 54.50 Mb | ORML2_CHICK | Un_random | 40.84 Mb | Contig436 | 39578 |
| DNAJC14 | 54.50 Mb | DNAJC14 | Un_random | 40.84 Mb | Contig436 | 39578 |
| RNF41 | 54.88 Mb | LOC426468 | Un_random | 3.14 Mb | Contig11101 | 1666 |
| ANR52_HUMAN | 54.92 Mb | ANR52_CHICK | Un_random | 40.25 Mb | Contig428 | 27215 |
| COQ10A | 54.95 Mb | LOC770970 | Un_random | 2.50 Mb | Contig10867 | 1118 |
| USP52 | 55.00 Mb | NP_001012972.1 | Un_random | 59.91 Mb | Contig876 | 12793 |
| ATP5B | 55.32 Mb | ATPB_CHICK | Un_random | 9.51 Mb | Contig13763 | 1111 |
| R3HDM2 | 55.93 Mb | R3HDM2 | Un_random | 42.03 Mb | Contig457 | 29464 |
| GLI1 | 56.14 Mb | GLI1_chicken | Un_random | 40.82 Mb | Contig436 | 39578 |

Among of the total potential orthologous proteins between HSA12 from 46 Mb to 60 Mb and the part of GGA1 (chicken chromosome 1), GGA2, GGA7 and ChrUn_random, which contained contigs that could not be localized to a chromosome or linkage group, 38 protein genes were not localized. Here is the list of these 38 genes.
